# Glial multicellular programs reveal distinct patient stratification in Parkinson's disease

**DOI:** 10.21203/rs.3.rs-9520754/v1

**Published:** 2026-04-30

**Authors:** Juan Manuel Barba-Reyes, Sergio Marco Salas, Gabriel Gonzalez-Ulloa, Lisbeth Harder, Nima Rafati, Maria Chatzinikolaou, Fabian J. Theis, Bradley T. Hyman, Alberto Serrano-Pozo, Mats Nilsson, Ana B. Muñoz-Manchado

**Affiliations:** 1 Unit of Cell Biology, Department of Neuroscience, Institute for Biomedical Research and Innovation of Cádiz (INiBICA), University of Cádiz, Cádiz, Spain; 2 Institute of Computational Biology, Helmholtz Center, Munich, Germany.; 3 Science for Life Laboratory, Department of Biochemistry and Biophysics, Stockholm University, Stockholm, Sweden; 4 Department of Information Technology, Uppsala University, Uppsala, Sweden; 5 Laboratory of Molecular Neurobiology, Department of Medical Biochemistry and Biophysics, Karolinska Institutet, Stockholm, Sweden; 6 National Bioinformatics Infrastructure Sweden, Uppsala University, SciLifeLab, Department of Medical Biochemistry and Microbiology, Uppsala, Sweden; 7 TUM, School of Computation, Information and Technology, Technical University of Munich, Germany. TUM School of Life Sciences, Technical University of Munich, Germany; 8 Department of Neurology, Massachusetts General Hospital, Boston Massachusetts, USA; 9 Harvard Medical School, Boston Massachusetts, USA; 10 Ciber de Salud Mental (CIBERSAM), ISCIII, Madrid, 28029 Madrid, Spain.

## Abstract

Parkinson’s disease (PD) is a neurodegenerative disorder characterized by nigrostriatal degeneration. While the role of glial cells in PD is increasingly recognized, the coordinated multicellular responses driving PD within the dorsal striatum remain poorly understood. By integrating single-nucleus RNA sequencing of 56 donors with targeted spatial transcriptomics, we disentangle regional from PD-related molecular programs across astrocytes, microglia and oligodendroglia. We identify PD-associated glial subpopulations organized into two distinct multicellular programs: one inflammatory and one UPR-associated, where each patient is dominated by one of these programs. Notably, these programs partition the molecular changes typically associated with PD into two specific, non-overlapping signatures. Multi-region analysis revealed these signatures are globally enriched across the sampled areas and Lewy body disease stages, from brainstem-predominant to neocortical, demonstrating that PD is characterized by mutually exclusive, brain-wide glial multicellular states. Our findings redefine glial alterations in PD as systemic and multicellular, providing a framework for patient stratification and the development of targeted, state-specific therapeutic interventions.

## Introduction

Parkinson’s disease (PD) is one of the most prevalent neurodegenerative disorders, affecting over 6 million people worldwide and being the fastest growing neurological disorder globally^1^. Clinically, PD manifests as a combination of motor symptoms such as bradykinesia, rigidity, and tremor, as well as non-motor deficits including cognitive impairment and mood disorders^2^. At the molecular level, PD is characterized by the aggregation of α-synuclein and other proteins into Lewy bodies (LB)^3^ and the subsequent progressive loss of dopaminergic neurons (DANs) in the substantia nigra pars compacta (SNpc), leading to striatal dopamine depletion and the above hallmark motor symptoms. Current therapies primarily aim to replace dopamine or reduce its catabolism to mitigate motor symptoms^1^, yet they do not halt disease progression, highlighting the urgent need to understand the mechanisms underlying cellular vulnerability.

Despite the central role of the SNpc in motor symptoms, PD involves diverse brain regions, with pathology typically spreading from the lower brainstem and olfactory bulb^4^, affected even decades before the onset of motor symptoms, to the midbrain, limbic structures, and neocortex, as described by the Braak LB staging^5^. While some studies report alterations across brain regions^6^, a substantial proportion of the studies focus on the midbrain, overlooking other relevant regions such as the dorsal striatum. In humans, the dorsal striatum comprising caudate nucleus (CN) and putamen (Pu), represents a hub for the integration of dopaminergic, glutamatergic, and cholinergic signaling and is the primary target of the nigrostriatal projections that degenerate in PD^7^. These properties make the dorsal striatum more than a passive receiver of dopaminergic input: it is where the functional consequences of nigral degeneration are expressed most directly, and where the motor and cognitive deficits that define PD ultimately originate^8,9^.

Significant progress has been made in characterizing the PD-associated molecular programs across cell types^6,10–15^. In this context, glial cells are increasingly recognized as central players^10^, as supported by transcriptomic studies reporting molecular dysregulations in glia^6,11,12,16,17^, coupled with the discovery that many PD-associated risk genes are predominantly expressed in non-neuronal cells^18^. Among the glia, astrocytes have shown PD alterations related to disrupted α-syn handling, impaired proteostasis and mitochondrial function, metabolic and calcium dysregulation, and pro-inflammatory reprogramming^13^, leading to toxic protein buildup and indirect neuronal damage. In the same line, microglia are thought to be one of the first cell types affected by the disease^19^. Diverse molecular pathways have been found activated in PD microglia, including unfolded protein response (UPR)^6,12,16^, as well as proinflammatory programs^6,12,20^. Oligodendroglia in PD share several pathological features with other glial cells^12,21^, such as inflammatory activation and stress responses. However, oligodendrocytes also exhibit unique alterations related to myelination and cell differentiation. Oligodendrocyte precursor cells (OPCs) additionally show impaired differentiation capacity potentially limiting their ability to replenish damaged myelin^14^. Beyond glia, other non-neuronal populations are increasingly implicated in the disease. For instance, emerging data indicates a breakdown of the neurovascular unit, where altered endothelial and pericyte activity compromises blood-brain barrier integrity^22^. While collectively these findings provide a comprehensive catalog of PD-associated molecular programs, the disease's inherent heterogeneity, prolonged clinical course, and diverse symptomatology, combined with the complex architecture of the dorsal striatum, leave significant knowledge gaps. It remains unclear how these alterations are coordinated across various cell populations or whether they function as primary drivers, secondary modulators, or compensatory responses. Furthermore, the extent to which these cellular programs are universal across all patients or restricted to specific clinical subsets remains to be determined.

In this study, we analyze glial transcriptomic changes in the dorsal striatum. To map these changes, we combined single-nucleus RNA sequencing and Xenium spatial transcriptomics, from 56 donors (54 CN and 56 Pu). We identify, not only the glia diversity but also, for the first time, distinct multicellular programs that drive patient stratification linked to specific pathological features. Furthermore, we demonstrate that this stratification applies beyond the dorsal striatum, defining a brain-wide, rather than regional, patient profile.

## Results

### 1. Transcriptomic single-nucleus analysis reveals distinct glial subpopulations in the dorsal striatum.

**Figure 1.**
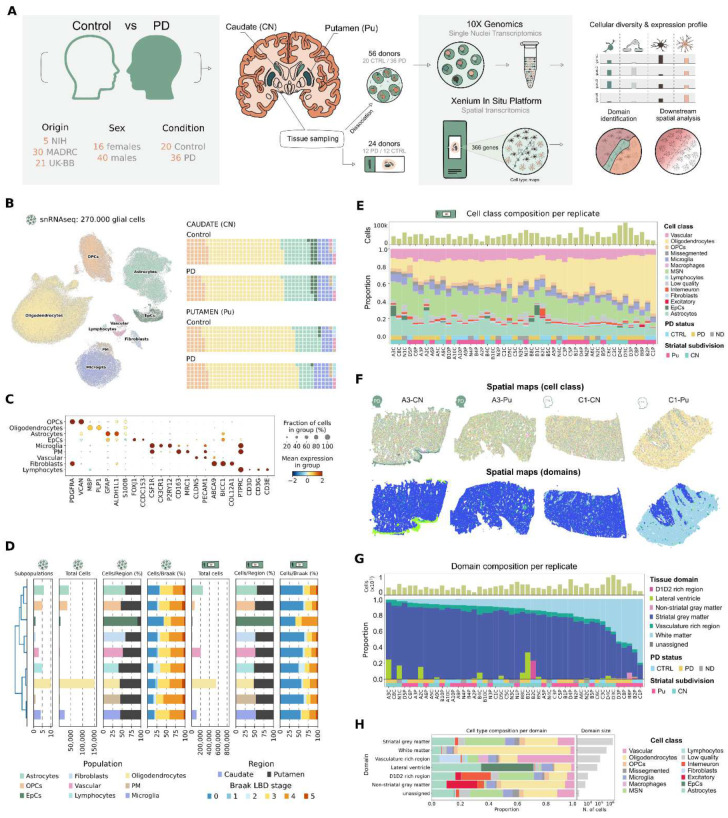
Study design and cellular diversity identified in the striatum **A.** Schematic representation of the experimental workflow followed to profile striatal samples using both snRNA-seq and spatial transcriptomics (Xenium) **B.** Glial diversity identified in the striatum using the snRNA-seq data, represented in the UMAP (left), with cellular frequencies of each cell type across PD status (PD, Controls) and regions profiled (Putamen, Caudate) (right). **C.** Markers for each population visualized in a dotplot. The size of the dot represents the % of cells expressing that gene in each cell type, and the colormap the mean expression in that group. **D.** Summary barplots for each identified cell population, showing from left to right: the number of subpopulations resolved within each major class; total cell counts retained after quality control in the snRNA-seq dataset; regional breakdown by CN and Pu; percentages of cells across Braak stages; total cell counts in the spatial transcriptomics dataset; regional breakdown in spatial data; and Braak stage distribution in spatial data. **E.** Cellular composition across Xenium-profiled samples. The stacked bar plot displays relative cell type proportions, while the upper bar plot indicates total cell counts per sample. Metadata, including PD status and anatomical region, are annotated below. **F.** Representative spatial maps of cells profiled using Xenium are shown, with cells colored by cell class (top) and by assigned domain (bottom). Cell class colors correspond to the palette shown in panel C. Four representative samples are displayed: two PD samples (caudate and putamen) and two control samples (caudate and putamen). **G.** Domain across Xenium-profiled samples. The stacked bar plot displays relative domain proportions, while the upper bar plot indicates total cell counts per sample. Metadata, including PD status and anatomical region, are annotated below. **H.** Compositional analysis of Xenium-defined tissue domains. Relative cell type frequencies are shown via stacked bar plot (left), with corresponding total cell yields per domain illustrated by the adjacent bar plot (right). *Icons used throughout the figures: the snRNA-seq gem miniplot indicates data derived from the single-nucleus RNA sequencing dataset; the tissue slide icon indicates data derived from the spatial transcriptomics dataset.

To reconstruct the cellular landscape of the human dorsal striatum in the context of PD, we profiled a large postmortem cohort of 56 individuals (20 neurotypical controls and 36 confirmed PD donors) (Fig. 1A, Extended Data Fig. 1, Extended Data Table 1) using single-nucleus RNA sequencing (snRNA-seq). We additionally performed image-based spatial transcriptomics (iST) using Xenium^23^ in a subset of 24 subjects (12 controls, 12 PD donors). For each donor, both CN and Pu sections were profiled using a 366-gene panel (Methods).

Following stringent quality control (see Methods section), our snRNA-seq dataset included ~600.000 high-quality nuclei, with ~270.000 identified as glial and non-neuronal cells, forming the basis of a detailed human striatal atlas (Fig. 1B–D). The cellular composition of this dataset reflected the compositional reality of glial cells in the striatum, largely dominated by oligodendrocytes, followed by astrocytes, oligodendrocyte precursor cells (OPCs), and microglia. We also captured critical low-abundance populations, including ependymal cells, vascular-associated cells, Fibroblasts, perivascular macrophages, and lymphocytes, ensuring a complete view of the neurovascular niche (Fig. 1B–D, Extended Data Table 2). This cellular diversity was also confirmed in the iST datasets, where we obtained the expression profile of ~2.337.000 cells, classified in 12 major cell classes (Fig. 1E, Extended Data Fig. 2A-C). We identified consistent subpopulations within each major cell class across both datasets (Fig. 1D, Extended Data Fig. 2D-G), with marked cellular heterogeneity within all glial and other non-neuronal populations. Importantly, these cell classes displayed comparable relative distributions across both regions and Braak LB stages (Fig. 1D). We further used module analysis^24^ to break down these complex expression profiles into distinct gene programs, uncovering both unique and shared cellular functions (Extended Data Figure 3).

Because the dorsal striatum is a heterogeneous structure, we next employed NicheCompass^25^ to identify tissue domains (Fig. 1F–G, see Methods). We identified two dominant domains: striatal gray matter (GM; 72.1% of cells), characterized by the presence of medium spiny neurons (MSN) (Fig. 1G), and white matter (WM; 19.0%), enriched in glial cells, particularly oligodendrocytes (Fig. 1G). In addition to these major domains, we detected regions corresponding to vasculature (5.1%), the lateral ventricle (2.8%) (Fig. 1F–G) and other rare sample-specific structures (<0.5%), including non-striatal grey matter (nsGM) and D1D2-rich regions. Domains exhibited a well-defined spatial organization, with WM regions located both surrounding and interspersed within the GM in the form of bundles (Fig. 1F). In addition to these bundles, the striatal GM was also embedded by vascular structures, enriched in immune and vascular cells. Finally, the lateral ventricle, marked by ependymal cells, was observed at the edge of the GM in a subset of samples, specifically adjacent to the CN (Fig. 1F–G), consistent with known neuroanatomical landmarks^26^.

### 2. Striatal astrocytes adopt distinct UPR-associated and reactive phenotypes in Parkinson’s disease

**Figure 2.**
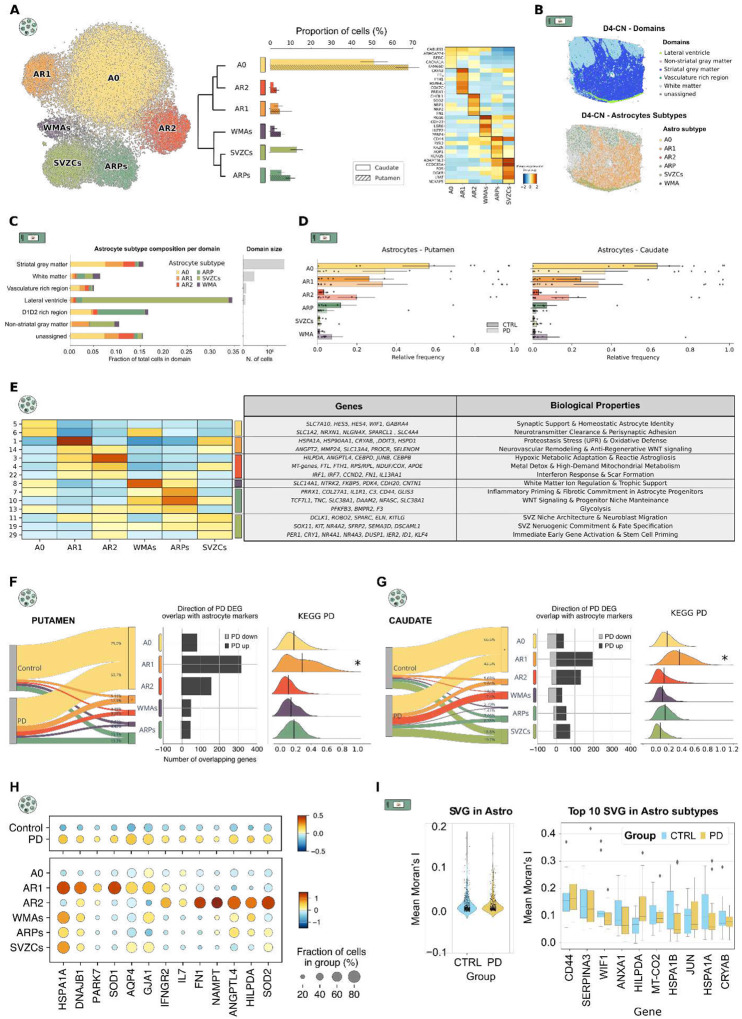
Analysis of astrocytic diversity in the striatum reveals two PD-associated subtypes **A.** Molecular and spatial characterization of astrocytes. The UMAP (left) visualizes astrocyte subtype clustering, while the middle bar plot shows their relative distribution in CN and Pu. The heatmap (right) highlights the expression profiles of the top five marker genes for each subtype, with the colormap representing the mean expression in each subpopulation. **B.** Comparative spatial mapping of a representative Xenium sample. The sample is visualized twice: first to show the tissue domain architecture (top) and second to highlight astrocyte subtype diversity (bottom). In the latter, astrocytes are color-coded by subtype against a background of gray-scaled “other” cell types. **C.** Stacked barplots showing frequencies of each subpopulation per domain identified in spatial data with corresponding total cells per domain illustrated by the adjacent bar plot (right). **D.** Compositional analysis of astrocytes clusters per condition in Xenium defined GM. Relative subpopulation frequencies in each condition are shown via barplots in both Pu (left) and CN (right). **E.** Subtype-specific enrichment of Hotspot modules in astrocytes subpopulations. This heatmap illustrates the average activity (module scores) of gene programs identified in astrocytes. Only modules showing enrichment in specific subtypes are shown, supplemented by a list of lead genes and their corresponding biological functions. **F-G.** Independent analysis of PD-associated astrocyte subpopulations in Pu (F) and CN (G). Each panel includes a river plot showing the relative abundance of each subtype across PD and Control groups (left), a stacked bar plot illustrating the overlap of PD differentially expressed genes (DEGs) with each subtype (middle), and the distribution of KEGG Parkinson’s Disease pathway scores across identified groups (right). **H.** Dotplot highlighting genes from DEA analysis overexpressed in PD that overlap with specific astrocytic subpopulations. **I.** Spatial gene expression variability in striatal gray matter astrocytes. Violin plots display the mean Moran’s I scores for all profiled genes, stratified by PD condition (left). This is complemented by a boxplot illustrating the distribution of scores for the top 10 genes with the highest mean Moran’s I values, grouped by clinical status (right).

We next focused on resolving astrocyte heterogeneity in PD and control striatum. We identified approximately 50,000 cells grouped into six transcriptionally distinct clusters (Fig. 2A, Extended Data Table 3). The largest subpopulation, Astrocytes 0 (A0; *CABLES1*, *RERG*, *CACNA1A*), represents homeostatic astrocytes and was predominantly enriched in controls, especially in GM domains (Fig. 2A–G). A0 showed high expression of synaptic support and astrocyte identity genes (*SLC7A10*, *HES4*, *HES5*, *WIF1)* (Fig. 2E, Extended Data Table 4). In addition, the elevated levels of neurotransmitter transporters and perisynaptic adhesion molecules (*SLC1A2*, *GPC5*, *NRXN1*, *NLGN4X*, *SPARCL1*) suggests a specialized role in glutamate clearance and stabilization of neuron-astrocyte contacts, in agreement with astrocyte’s role in synaptic homeostasis (Fig. 2E, Extended Data Table 4).

In contrast to A0, we identified two astrocytic reactive (AR) states, namely AR1 and AR2. Both AR subpopulations showed increased abundance in PD, although AR1 reached statistical significance only in Pu (p < 0.05) (Fig. 2A–B, 2D, 2F–G). While both AR states were enriched in GM, they presented distinct functional specializations (Fig. 2B, 2C). AR1^12^ (*CRYAB*, *FTH1*, *HSPA4L*) was characterized by robust proteostasis and oxidative stress responses pathways, marked by the upregulation of unfolded protein response (UPR) and heat shock-related genes (*HSPA1A*, *HSP90AA1*, *CRYAB*, *DDIT3*, *HSPD1*) (Fig. 2E, Extended Data Table 4). AR1 also exhibited transcriptional signatures of neurovascular remodeling and anti-regenerative WNT-related processes, with elevated expression of *ANGPT2*, *MMP24*, *SLC13A4*, *PROCR* and *SELENOM* (Fig. 2E, Extended Data Table 4).

AR2 (*CHI3L1*, *FN1*, *SOD2*) represented a more immunologically active and metabolically adapted reactive state. This subpopulation was defined by a signature of reactive astrogliosis, overexpressing hypoxia-associated genes (*HILPDA, ANGPTL4*) and key regulatory transcription factors (*CEBPD,JUNB,CEBPB*) (Fig. 2E, Extended Data Table 4). To counter iron-mediated toxicity and energetic failure, AR2 upregulated iron-storage ferritins (*FTL*, *FTH1*) and key components of the mitochondrial respiratory chain (NDUF and COX families) (Fig. 2E, Extended Data Table 4). Inflammatory and tissue-remodeling signatures were also evident, characterized by the upregulation of lipid-transport apolipoproteins (*APOE*, *APOC1*) and interferon-regulatory factors (*IRF1*, *IRF7*). This profile, coupled with increased levels of the cell-cycle regulator *CCND2*, the matrix protein *FN1*, and the signaling receptor *IL13RA1*, indicates a shift toward interferon-mediated responses and the structural deposition required for glial scar formation (Fig. 2E, Extended Data Table 4).

AR1 and AR2 were also identified as the primary astrocytic drivers of transcriptomic reprogramming in PD, showing the strongest overlap in expression with astrocyte-specific DEGs in PD. (Fig. 2F–G, Methods). In both Pu and CN, AR1-associated DEGs reflected a proteostatic and antioxidant response described by elevated *HSPA1A,DNAJB1,PARK7,* and *SOD1* (Fig. 2H). Conversely, AR2-associated DEGs presented a pro-inflammatory profile, marked by immune-related genes (*IFNGR2,IL7*), extracellular matrix remodeling factors (*FN1*), and metabolic stress regulators (*NAMPT,ANGPTL4,HILPDA*), together with elevated mitochondrial stress signaling through *SOD2*^27^ (Fig. 2H). In addition, pathway enrichment scores associated with KEGG PD ontology^28^ revealed significant enrichment within AR1 cells in both regions (Pu: LFC = 0.77, p-value = 5.84 × 10^−55^; CN: LFC = 1.39, p-value = 0) (Fig. 2F, 2G, Extended Data Table 5), linking this stress-responsive state to established PD-related molecular hallmarks.

Beyond the GM-associated subpopulations, two astrocytic subpopulations were enriched in WM. White Matter Astrocytes^29^ (WMA; *RGS6*, *LGR5*, *LUZP2*) expressed genes associated with solute transport and metabolic adaptation (*SLC14A1*, *FKBP5*, *PDK4*) alongside axonal support and adhesion molecules (*NTRK2*, *CDH20*, *CNTN1)*, consistent with a role in WM integrity (Fig. 2A, 2E, Extended Data Table 4). Astrocyte Restricted Precursors^30^ (ARP; *CD44*, *RYR3*, *KAZN*) represented a progenitor-like state combining astrocytic identity with inflammatory priming and early fibrotic commitment, marked by extracellular matrix and injury-associated genes (*PRRX1,COL27A1,TNC,GFAP,VIM*), inflammatory mediators (*IL1R1,C3,F3*), and developmental regulators (*TCF7L1,GLIS3,DAAM2,BMPR2*). Neither subpopulation was strictly confined to WM (Fig. 2A, 2E, Extended Data Table 4).

Finally, we identified a subpopulation corresponding to subventricular zone cells (SVZCs; *ADAMTSL3*, *CCDC85A*, *FOS*), restricted to the lateral ventricle domain and therefore exclusively observed in CN samples (Fig. 2A, 2C). SVZCs showed a transcriptional program consistent with SVZ niche organization and neuroblast migration^31^, with enrichment for extracellular matrix and guidance genes (*SPARC,ELN,ROBO2,SEMA3D,DSCAML1*), neurogenic commitment markers (*KIT,KITLG,SOX11,SFRP2,NR4A2*), and an immediate early gene signature (*NR4A1,NR4A3,DUSP1,IER2,ID1,KLF4*) (Fig. 2E, Extended Data Table 4).

Spatial autocorrelation analysis (Moran's I) of the iST dataset showed consistently low scores across all profiled genes (I < 0.2) in both conditions, indicating that spatial organization is not a primary driver of striatal astrocytic heterogeneity in health or PD (Fig. 2I)

### 3. Striatal microglia exhibit distinct pro-inflammatory and UPR-associated phenotypes in Parkinson’s disease

**Figure 3.**
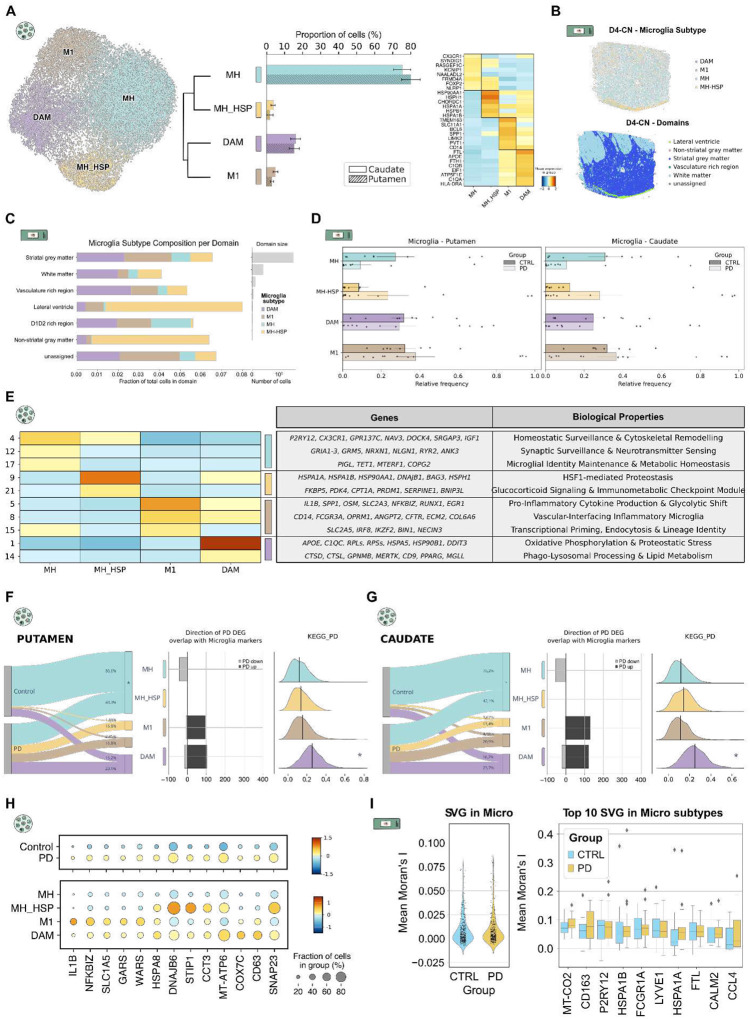
Analysis of microglial diversity in the striatum reveals two PD-associated subtypes **A.** Molecular and spatial characterization of microglia. The heatmap (right) highlights the expression profiles of the top five marker genes for each subtype, while the middle bar plot shows their relative distribution in CN and Pu. The UMAP (left) visualizes microglial subtype clustering. **B.** Comparative spatial mapping of a representative Xenium sample. The sample is visualized twice: first to show the tissue domain architecture (top) and second to highlight microglial subtype diversity (bottom). In the latter, microglia are color-coded by subtype against a background of gray-scaled “other” cell types. **C.** Stacked bar plots showing the relative abundance of each microglial subpopulation within each spatially defined tissue domain. Total cell counts per domain are shown in the adjacent bar plot. **D.** Relative abundance of microglial subpopulations in control and PD conditions, restricted to gray matter as defined by Xenium spatial data, shown separately for Pu (left) and CN (right). **E.** Subtype-specific enrichment of Hotspot modules in microglia. This heatmap illustrates the average activity (module scores) of gene programs identified in microglia. Only modules showing enrichment in specific subtypes are shown, supplemented by a list of lead genes and their corresponding biological functions. **F-G.** Independent analysis of PD-associated microglial subpopulations in the Putamen (F) and Caudate (G). Each panel includes a river plot showing the relative abundance of each subtype across PD and Control groups (left), a stacked bar plot illustrating the overlap of PD differentially expressed genes (DEGs) with each subtype (middle), and the distribution of KEGG Parkinson’s Disease pathway scores across identified groups (right). **H.** Dotplot showing PD-associated differentially expressed genes with enriched expression in specific microglial subpopulations. **I.** Spatial gene expression variability in striatal gray matter microglia. Violin plots display the mean Moran’s I scores for all profiled genes, stratified by PD condition (left). This is complemented by a boxplot illustrating the distribution of scores for the top 10 genes with the highest mean Moran’s I values, grouped by clinical status (right).

Microglia also showed high molecular diversity, with transcriptional states tightly coupled to PD progression^11,12,32^. To characterize these shifts, we analyzed the approximately 30,000 cells in our single-nuclei dataset, divided into four subpopulations. Homeostatic microglia (MH) (*CX3CR1,SYNDIG1,KCNIP1*) represented 70–80% of all microglia in both Pu and CN (Fig. 3A, Extended Data Table 6). Their transcriptional profile reflected active environmental surveillance (*P2RY12,CX3CR1*), cytoskeletal dynamics (*NAV3,DOCK4,SRGAP3*), synaptic monitoring (*GRIA1–3,GRM5,NRXN1,NLGN1,RYR2,ANK3*), and metabolic stability (*IGF1, TET1*), consistent with long-term homeostatic maintenance rather than immune activation (Fig. 3E, Extended Data Table 7). MH cells were significantly decreased in PD in both Pu and CN (p = 0.013 and p = 0.042, respectively) (Fig. 3D, 3F, 3G).

Mirroring our astrocyte findings, two microglial subpopulations were overrepresented in PD: pro-inflammatory Microglia (M1) and Heat Shock Protein-responsive Microglia (MH-HSP) (Fig. 3A, 3B, 3D–G). M1 cells (*TMEM163,SLC11A1,BCL6*) showed elevated inflammatory mediators (*IL1B, SPP1, OSM*), glycolytic transporters (*SLC2A3, SLC2A5*), immune activation markers (*NFKBIZ,RUNX1,EGR1,IRF8,IKZF2*), myeloid identity genes (CD14, FCGR3A, BIN1), and vascular-interfacing factors (*ANGPT2,ECM2,COL6A6*), consistent with a shift toward high-demand inflammatory metabolism and vascular interaction (Fig. 3E, Extended Data Table 7). In PD, M1 cells showed further upregulation of *IL1B,NFKBIZ*, amino acid transporters (*SLC1A5*), and tRNA synthetases (*GARS*, *WARS*), reflecting the elevated biosynthetic demands of active neuroinflammation (Fig. 3H).

The second PD-enriched subpopulation, MH-HSP, mirrored the AR1 astrocyte profile, with activation of proteostasis and stress-adaptation programs (Fig. 3A, 3B, 3D, 3F, 3G). MH-HSP cells showed elevated expression of HSF1-target heat shock genes (*HSPA1A, HSPA1B, HSP90AA1, DNAJB1, BAG3, HSPH1*), glucocorticoid-responsive metabolic regulators (*FKBP*5, TSC22D3, PDK4, CPT1A), immune modulators (*PRDM1, PELI1, SERPINE1*), and autophagy-related genes (*ULK1,BNIP3L*) (Fig. 3E, Extended Data Table 7). Although MH-HSP was more abundant in PD, this trend did not reach statistical significance (Fig. 3D, 3F, 3G).

Disease-associated microglia (DAM; *FTL,APOE,FTH1*) were the second most abundant microglial population (Fig. 3A). Their profile combined oxidative phosphorylation and proteostasis genes (*COX7C, ATP5F1E,NDUFS5,HSPA5,HSP90B1,DDIT3*), elevated ribosomal components (*RPLs, RPSs*), phago-lysosomal and lipid metabolism markers (*CTSD,CTSL,GPNMB,MERTK,CD9,APOE,PPARG,MGLL*), and complement genes (*C1QC*), consistent with established DAM signatures^33^ reflecting active debris clearance under metabolic stress (Fig. 3E, Extended Data Table 7). Despite a non-significant compositional increase in PD, KEGG PD ontology scoring revealed significant enrichment in DAM in both regions (Pu: LFC = 0.96, p_adj_ = 0; CN: LFC = 0.97, p_adj_ = 0) (Extended Data Table 8), directly linking this state to PD-related molecular pathways (Fig. 3F, 3G). In PD, DAM showed a pronounced proteostatic signature driven by *HSP70/HSP90* co-chaperones (*HSPA8, DNAJB6, STIP1*), chaperonins (*CCT3*), mitochondrial transcripts (*MT-ATP6, COX7C*), and vesicle trafficking genes (*CD63, SNAP23*) (Fig. 3H).

Microglial composition was largely consistent across domains, with the exception of the lateral ventricle, which showed strong MH-HSP enrichment (Fig. 3C), likely reflecting the inherently stress-prone nature of this niche, where proteostatic programs are active even under homeostatic conditions. As observed for astrocytes, spatial autocorrelation analysis of Xenium data showed consistently low Moran's I scores (<0.2) across all profiled genes in GM, indicating that spatial organization is not a primary driver of striatal microglial heterogeneity in either health or PD (Fig. 3I).

### 4. Vascular and immune alterations during PD in the striatum

To complete the characterization of striatal glial diversity, we integrated the oligodendroglial lineage (oligodendrocytes and OPCs) profiled in detail in another study^14^, which resolved 15 subpopulations including developmental states and PD-associated (PDA) clusters in both oligodendrocytes (PDAOs) and OPCs (PDAOPCs). These PD-enriched subpopulations shared molecular programs with the stress-responsive states identified in astrocytes (AR1) and microglia (MH-HSP). Their classifications were incorporated into the present study to enable the multicellular analyses described below.

Beyond glial cells, we also identified cellular diversity within other non-neuronal populations. For instance, vascular cells could be divided into five subpopulations, including endothelial cells (*CLDN5,PTPRB*), fibroblasts (*FLRT2,ABCA9,ATP1A2*), vascular leptomeningeal cells (VLMCs; *SAT1, THBS1, PTPRG, CYP1B1*), pericytes (*PDE8B, RGS5,PDGFRB*), and vascular smooth muscle cells (VSMCs; *MYH11,MCAM,PDE3A*). Among these, we only identified VSMCs to be significantly enriched in control subjects in CN, suggesting a disease-associated decrease of this population in PD (Extended Data Fig. 4).

Finally, lymphocytes represented the least abundant population in our dataset. These cells were categorized into five distinct subpopulations: CD4+ memory T cells (CD4_TM; *LEF1, PRKCA*), CD8+ tissue-resident memory effector cells (CD8_TRME; *MVB12B,IL7R,GNLY, PLEK*), CD8+ tissue-resident memory T cells (CD8_TRMM; *PARP8,PATJ,BCL2*), B cells (*ARHGAP24,MEF2C,CDK14*), and NK cells (*KLRF1,PDGFD,CD247,NKG7*) (Extended Data Fig. 4). Despite their low abundance, the presence of these subpopulations within the striatal GM aligns with recent evidence of peripheral immune cell infiltration in PD^34^.

### 5. Glial stratification of PD donors reveals two mutually exclusive multicellular programs

While previous studies^6,11,12,14,15^ and our results link PD to widespread transcriptomic shifts in non-neuronal populations, small cohort sizes have largely limited these findings to identifying associations between individual molecular programs and a diagnosis of PD. However, PD is a substantially heterogeneous disorder^35,36^, characterized by a complex interplay of clinical trajectories, pathological burdens and multifaceted molecular landscapes.

**Figure 4.**
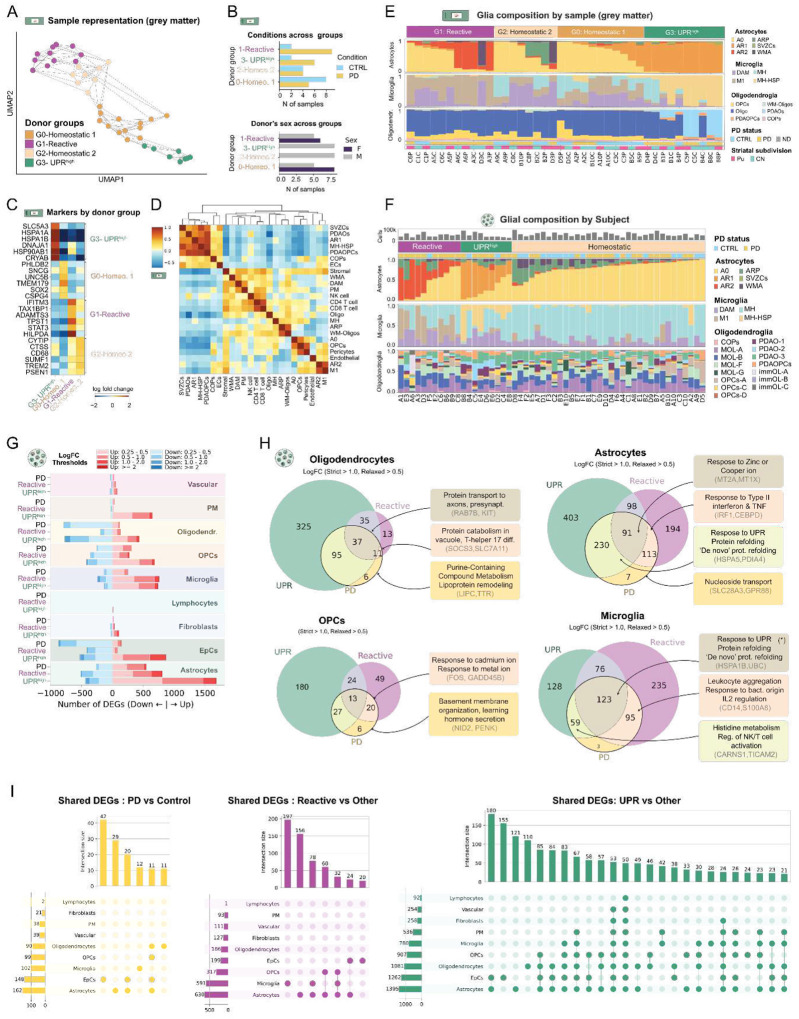
PD-associated multicellular programs identified by compositional analysis of glial subpopulations in striatum. **A.** Spatial representation of Xenium datasets. The map includes only cells identified within the striatal gray matter domain, with individual cells color-coded by their assigned donor group. **B.** Sample composition by donor group. The upper bar plot illustrates the distribution of samples based on PD status (top), while the lower bar plot provides a breakdown by donor sex (bottom). **C.** Differential expression across patient groups. This heatmap displays the mean expression levels for the top six differentially expressed genes (DEGs) identified for each donor group, compared across all cohorts. **D.** Correlation of cellular architecture in the striatal gray matter. The heatmap illustrates the Pearson correlation of pairwise cell subtype abundances across all Xenium samples, restricted to cells within the gray matter domain. **E.** Cell type-specific subtype composition in the striatal gray matter. The stacked bar plot illustrates the relative proportions of astrocyte, microglia, and oligodendroglia subtypes across Xenium samples. To provide context for each sample, the total cell count is indicated in the upper bar plot (top), while corresponding metadata, including PD status and donor group, are annotated directly above the compositional data. **F.** Cell type-specific subtype composition in snRNA-seq samples. The stacked bar plot illustrates the relative proportions of astrocyte, microglia, and oligodendroglia subtypes across snRNA-seq samples. The total cell count for each sample is indicated in the upper bar plot (top), while corresponding metadata, including PD status and donor group, are annotated directly above the compositional data. **G.** Differential gene expression (DGE) analysis across PD-associated states. The stacked bar plot illustrates the count of upregulated and downregulated genes across each cell type for three comparisons: PD vs. Control, UPR^high^, vs. homeostatic, and Reactive vs. homeostatic. Significance is defined by an adjusted $p$-value $< 0.05$ and $|\log_{2}FC| > 0.25$. Genes within each bar are further stratified by their specific fold-change magnitude. **H.** Transcriptional overlap across disease states. Venn diagrams depict shared and unique DEGs between PD, UPR^high^, and Reactive groups for astrocytes (top, left), OPCs (top, right), oligodendrocytes (bottom, left), and microglia (bottom, right). Annotations highlight significant enriched pathways and representative genes within key intersections. **I.** Differential expressed genes shared across cell types. Upset plots illustrate the number of differentially expressed genes (both upregulated and downregulated) shared among cell types when comparing PD vs. Control (right=, UPR^high^, vs. homeostatic (middle), and Reactive vs. homeostatic (right). Only intersections containing more than 10 genes were visualized

We next focused on exploring donor heterogeneity. Importantly, due to striatum’s architecture, we anticipated that inconsistent sampling of WM or ventricular space alongside GM would act as a primary driver of molecular variance, potentially masking true pathological signals. To mitigate this cofounder, we first assessed donor variability using iST datasets, where we can consider only cells located in the GM. Using Gloscope^37,38^, we identified four distinct groups of donors. Although all groups included both controls and PD samples, Homeostatic 1 and 2 groups were strongly enriched in healthy donors, while UPR-high and Reactive groups were enriched in PD donors (Fig. 4A, 4B, 4C). Consistently, the two PD-enriched groups showed a higher abundance of glial subpopulations overrepresented in PD (Fig. 4D, 4E, Extended Data Fig. 5A-C). Cellular compositions of these groups were non-overlapping (Fig. 4D, 4E), suggesting a glial stratification of PD patients into distinct mutually exclusive multicellular programs. To confirm these findings, we extended the stratification analysis to snRNA-seq data by classifying donors into Homeostatic, Reactive, or UPR-high groups based on their cellular composition (see Methods). Despite the lack of spatial architecture in this dataset, the snRNA-seq donor groups exhibited cellular compositions highly consistent with the spatial results (Fig. 4F, Extended Data Fig. 5D-G).

The UPR-high donor group was found only in male donors (Fig. 4B) and was characterized by a high abundance of UPR-related subpopulations (AR1, MH-HSP, PDAOPCs, PDAOs) which covaried strongly across samples, suggesting coordinated multicellular regulation (Fig. 4D, 4E). In UPR-high related samples, these subpopulations accounted for nearly all cells of a given type, indicating population-wide activation. Analysis of the three canonical UPR branches (IRE1α, ATF6, and PERK^39^) did not point to a single dominant pathway, but suggested cell-type preferences: elevated MAPK8 in astrocytes, consistent with IRE1α signaling, and higher ATF6 expression in microglia (Extended Data Fig. 7).

In contrast, the Reactive donor group was defined by a high abundance of AR2 astrocytes and M1 microglia, which showed independent but complementary regulatory programs (Extended Data Fig. 4). M1 microglia were enriched for innate immune effector regulons (NF-κB, IRF8, STAT2, BACH1) and phagocytic remodeling (TFEC), while AR2 scored high for regulons associated with stress-adapted cellular identity (HES1, RFX4), metabolic adaptation (SREBF1, EPAS1, FOXO1), and an IFN-γ-biased inflammation (STAT1, ARID5A) (Extended Data Fig. 4). Despite these distinct profiles, both subpopulations covaried strongly across samples (Fig. 4C, Extended Data Fig. 5E), pointing to a coordinated multicellular program. Given that astrocytes and microglia are known to collaboratively participate in brain homeostasis and drive pathological^13,40,41^, we next performed cell-cell interaction analysis between AR2 and M1 (Extended Data Fig. 7)^42,43^. Our analysis revealed prominent crosstalk through the complement-mediated C3-CD81 axis, damage-associated HMGB1-TLR4 signaling, lipid and lysosomal pathways (APOE-ABCA1, PSAP-LRP1/SORT1), and fate-modulating interactions (SEMA4D-PLXNB1, CNTN1-NOTCH1) (Extended Data Fig. 7). These interactions define a feed-forward astrocyte-microglia network coupling inflammation, metabolic strain, and impaired clearance in the PD striatum.

Since many PD-DEGs were predominantly expressed in subpopulations specific to individual donor groups, we asked whether PD-DEGs reflect group-specific responses or a shared disease program. Cell-type-specific differential expression analysis comparing each donor group against homeostatic controls, contrasted with the global PD-versus-control analysis, allowing us to separate core disease hallmarks from group-specific signatures (Fig. 4G, Extended Data Fig. 6). In every cell type, the number of DEGs within each donor group substantially exceeded those from the global comparison. Microglia and astrocytes showed the highest transcriptomic burden, but notable shifts were also observed in oligodendroglia, perivascular macrophages (with many upregulated genes in the UPRhigh group), and ependymal cells, which showed widespread upregulation in the UPR-high group alongside broad downregulation across both groups (Fig. 4G).

Next, we compared the PD-specific with the donor group-specific DEGs (Fig. 4H), identifying a high proportion of the PD-DEGs found to be differentially expressed in only one of the donor groups. In astrocytes, for instance, 79.1% (343/441) of PD-DEGs were DEGs in only one of the two PD-associated donor groups, with only a small fraction found of the genes identified as DEGs in all donor groups, or restricted to PD. Subsequently GSEA confirmed that each of these distinct gene subsets were enriched for specific molecular functions.

DEGs shared between PD and the UPR-high group were broadly distributed across glial cell types (Fig. 4I) and enriched for cellular survival and UPR repair programs (Fig. 4H, Extended Data Fig. 6). Microglia-specific genes in this group were involved in histidine catabolism (producing antioxidants or histamine to suppress TNF-α under proteotoxic stress), while oligodendroglial genes were enriched for Cajal body functions, suggesting increased spliceosome assembly to repair stress-damaged transcripts (Fig. 4H).

In contrast, DEGs shared between PD and the Reactive group captured the neurotoxic and immune-interactive components of disease, concentrated in microglia and astrocytes with some extension to OPCs (Fig. 4I). Astrocytes were defined by Type II interferon, *TNF*, and *p38 MAPK* pathway activation, while microglia showed enrichment for leukocyte aggregation genes (*ICAM-1,VCAM-1*), consistent with physical engagement of infiltrating T cells (Fig. 4H).

Importantly, a core set of DEGs shared across both PD-associated groups likely reflects general disease features. Astrocytes were enriched for metal response genes (zinc, copper, iron), consistent with their role as CNS metal gatekeepers and suggesting ferroptosis prevention as a shared baseline response. Oligodendrocytes showed enrichment for protein transport to protrusions. Notably, microglia showed proteostatic stress and chaperone-dependent folding genes across all three groups, consistent with their constitutive burden of extracellular α-syn clearance regardless of activation state (Fig. 4H).

Finally, a small subset of genes differentially expressed in PD but not in either donor group, potentially reflecting features of homeostatic PD donors, included nucleoside transport in astrocytes (consistent with a ρ0-like metabolic shift^44^), purine metabolism and lipoprotein remodeling in oligodendrocytes, basement membrane organization in OPCs, and fibroblast organization regulation in ependymal cells (Fig. 4H).

Altogether, our findings indicate that the striatal PD signature is a composite of distinct glial pathological states, where a shared core of cellular stress is layered with specialized transcriptomic programs that define the transition from a homeostatic baseline to either a shared or cell-type-specific diseased environment.

### 6. Systemic activation of divergent glial multicellular programs in PD

**Figure 5.**
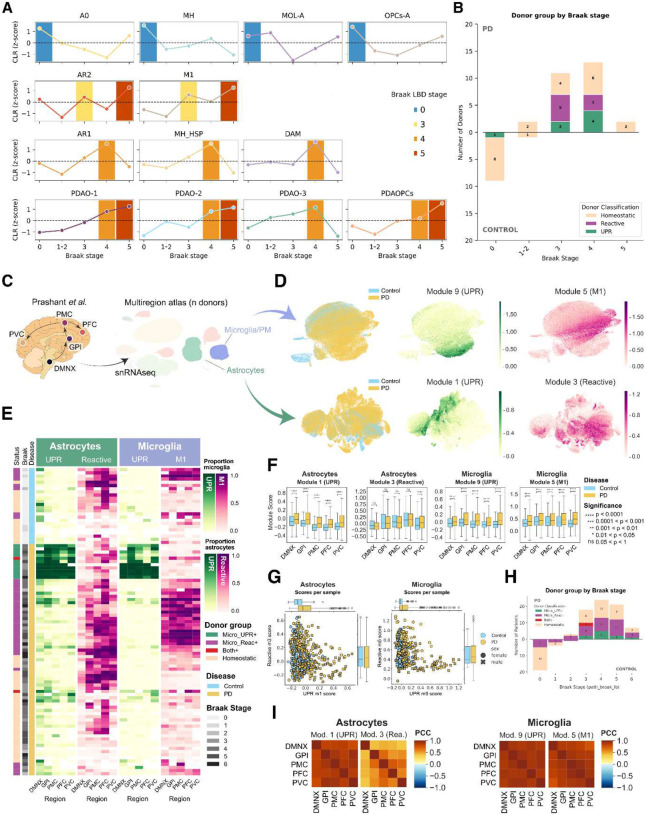
Braak stage-dependent dynamics and regional distribution of glial molecular programs in Parkinson’s disease **A.** Lineplots showing CLR-transformed abundance of each subpopulation across Braak stages, grouped by the molecular program to which each subpopulation belongs. **B.** Barplots showing the number of donors at each Braak stage, stratified by molecular program and condition (PD, upper panels; controls, lower panels). **C-D.** Glial subtype signatures in the multiregional PD atlas. Overview of the multiregion atlas (Prashant *etal*.) highlighting the isolation of microglia and astrocytes (C). Accompanying UMAP plots visualize subpopulations by disease status, UPR activation, and reactive/M1 signatures (D). **E.** Distribution of disease-associated glial phenotypes across donors and brain regions. Heatmap depicting the frequency of microglial and astrocytic subtypes within each profiled region per donor. Rows represent individual donors, annotated by clinical metadata (Braak stage, PD status, and donor group) on the left sidebar. **F.** Enrichment of PD-associated signatures by disease status. Box plots illustrate the distribution of module scores across microglial and astrocytic subtypes, stratified by PD status. Quantifications for all signature modules are presented for each profiled anatomical region. **G.** Correlation between UPR and reactive signatures in microglia. Scatter plot illustrating the relationship between mean UPR^high^ (x-axis) and Reactive/m1 (y-axis) microglial (right) and astrocytic (left) signature scores across all samples from Prashant et al. Individual data points are colored by donor sex and shaped by PD status (e.g., circles for Control, triangles for PD). **H.** Barplots showing the number of donors at each Braak stage, stratified by molecular program and condition (PD, upper panels; controls, lower panels). **I.** Regional correlation of cell-type signature scores. Heatmap displaying pairwise Pearson correlations of mean signature scores across all donor samples for Astrocytes (left) and Microglia (right).

Next, we assessed whether these multicellular programs align with disease progression, using Braak LB stages as a proxy (Fig. 5A). As expected, homeostatic subpopulations (A0, MH, MOL-A, OPCs-A), were most abundant at BS 0 and declined as neuropathological burden increased, reflecting a progressive loss of basal glial support. In contrast, subpopulations related with the two PD-associated programs exhibited an alternating dominance rather than a sequential progression. Importantly, both PD-associated donor groups were represented across Braak LB stages (Fig. 5A–B). Subpopulations related to the reactive donor group (AR2, M1) peaked at BS 3 and 5, whereas the UPR-associated subpopulations (AR1, MH-HSP, PDAO/PDAOPCs) became most prominent at BS 4. This pattern across stages argues against a linear transition from one program to the other, suggesting instead that the two programs operate as distinct responses across individuals rather than a fixed temporal sequence. To determine if these two PD-associated multicellular programs map onto established pathological axes, we evaluated their correlation with LB burden using previously quantified immunohistochemical measures in a subset of donors (Extended Data Fig. 5B^14^). Remarkably, no strong positive correlation was found between any PD-associated subpopulation and LB density, with only PDAOPCs and AR1, presenting weak correlations in both Pu (r = 0.5, p = 0.069 and r = 0.42, p = 0.134, respectively) and CN (r = 0.46, p = 0.131 for PDAOPCs).

Since other brain regions have previously reported similar glial responses in PD^12,17,18,45^, we hypothesized that our findings might apply beyond the dorsal striatum, potentially representing brain-wide molecular programs rather than a region-specific phenomenon. To explore this, we applied module scoring for our UPR-high and Reactive/M1 signatures (Fig. 2E, 3E) to a multi-region PD-atlas generated using snRNA-seq dataset^46^, comparing areas along the pathological continuum: from early-affected (DMNX; dorsal motor nucleus of the Xth nerve; GPi: globus pallidus interna) to late-affected (PMC: primary motor cortex; DLPFC: dorsolateral prefrontal cortex) and relatively spared (PVC: primary visual cortex) tissues (Fig. 5C). Across nearly all regions, PD cells scored significantly higher than control cells for the analyzed modules (Fig. 5D–F), with the exception of the reactive astrocyte signature (Module 3), which exhibited enrichment in PD cells only within the PVC (Fig. 5F). Remarkably, as we observed in the striatum, the UPR and Reactive signatures remained negatively correlated across donors (Fig. 5E). In addition, UPR-high signatures for both microglia and astrocytes co-varied across donors, as well as between M1 microglia and reactive astrocytes, suggesting that the multicellular programs described in the striatum are, to a large extent, conserved across brain regions (Fig. 5E).

To distinguish between transient stages and stable patient stratification, we examined cross-regional patterns within individual donors. If these signatures represented progressive waves, we would expect a sequential shift from early-affected regions (DMNX, GPi) toward the neocortex. Instead, signatures remained remarkably consistent across all regions within each donor (Fig. 5E, 5F). Leveraging the transcriptomic homogeneity of microglia, we classified donors as Reactive (>10% M1), UPR-high (>10% UPR), Homeostatic (both <10%), or Mixed (both >10%) (Fig. 5E, 5G). Notably, only two of 100 donors exhibited a mixed phenotype, reinforcing the existence of distinct, mutually exclusive molecular states. Comparison of these molecular groups against Braak LB stage revealed that while UPR-high donors tended toward later stages, both subtypes spanned the entire pathological spectrum. Notably, UPR-high cells occupied regions typically devoid of LBs in intermediate-stage (BS III-IV) donors (Fig. 5G). This suggests the UPR response is independent of local protein aggregation, potentially driven by soluble α-synuclein or a brain-wide, non-cell autonomous mechanism. As observed in the striatum, the UPR-high phenotype remained significantly overrepresented in male donors (Fig. 5G).

## Discussion

In this study, we integrated snRNA-seq (~270,000 nuclei) with high-resolution spatial transcriptomics (iST) to generate a comprehensive cellular atlas of the human striatum and dissect PD-associated molecular programs. The intermingled architecture of the dorsal striatum makes spatial context a biological necessity rather than a technical refinement. By delineating discrete vascular, ventricular, and gray/white matter niches, we avoided the signal homogenization and sampling bias inherent to bulk or dissociated single-cell studies. This precise spatial control allowed us to ensure that the transcriptomic profiles reflect authentic cellular reprogramming of specific tissue domains rather than sampling differences, unmasking glial responses that would otherwise be lost to the noise of striatal heterogeneity.

A central part of the study was the characterization of astrocytes and microglia, which revealed functionally distinct subpopulations. Critically, the reactive states identified in each population were not redundant. In astrocytes, AR1 and AR2 captured proteostatic and inflammatory responses respectively, while in microglia, KEGG scoring identified DAM rather than M1 as most strongly aligned with canonical PD pathways, suggesting that proteostatic load is an important defining feature.

When analyzing subpopulation co-occurrence across samples, we identified two independent multicellular programs that stratify PD donors into UPR-high, Reactive, or Homeostatic groups. While cell type-specific signatures within these programs have been linked to PD before^6,11,47^, even specifically in the dorsal striatum^48^, our data reveals for the first time that they form coordinated, mutually exclusive molecular signatures. The Reactive program represents a synchronized neuroinflammatory axis between microglia and astrocytes, a partnership increasingly recognized in neurodegeneration^33,41,49^. In contrast, the UPR-high program defines a distinct pathological state. Consistent with previous reports in the Pu^16^ and prefrontal cortex^50^, this UPR signature is characterized by widespread proteostatic stress markers and, notably, a lower burden of LBs, a finding that aligns our striatal data with broader cortical observations^50^. Many DEGs within these donor groups are lost in standard PD-vs-Control comparisons, as these signatures are restricted to specific subsets and their signals are diluted during aggregated analysis. In contrast, other programs appear to be present across PD-donors. For instance, metal detoxification in astrocytes, which have also been reported to happen in prefrontal cortex^50^.

By extending our analysis to a multi-region atlas^46^, we found that the UPR and Reactive profiles are not localized alterations but global activation patterns, showing high intra-donor consistency across the brain. This is particularly significant for the UPR-high signature: while the UPR is traditionally viewed as a local reaction to protein aggregation, we observed it in regions entirely lacking LB pathology, even in low Braak LB-stage donors. Our data suggests that PD-associated UPR activation is a brain-wide, potentially non-cell-autonomous phenomenon across glial networks. While such coordinated activation is a hallmark of viral brain infections^51^, evidence for a similar mechanism in neurodegeneration remains limited^52^. Further research is now required to uncover the specific molecular drivers of this brain-wide glial synchrony.

Both multicellular programs were found across donors from all Braak LB stages. However, we identified subtle differences, with UPR-high donors presenting overall a higher Braak LB stage. In addition, while the Reactive phenotype showed no sex bias, the UPR-high signature was significantly enriched in men. This sex-specific divergence in the glial response is an intriguing finding that warrants further investigation, given that PD is twice as frequent in men than in women^53,54^.

Despite these advances, several limitations should be noted. First, our snRNA-seq dataset and high-resolution spatial transcriptomics data is restricted to the dorsal striatum, meaning the cellular niches and glial subpopulations defined here, including the AR1/AR2 astrocyte states and DAM microglia, were characterized within a single brain region. Whether equivalent spatial organization and reactive subpopulations exist in other areas remains to be directly tested. Second, while cross-referencing with a multi-region transcriptomic atlas suggests that the UPR-high and Reactive programs extend beyond the striatum, this extrapolation relies on an external dataset and therefore represents a convergent observation rather than direct validation. Third, while we observed a significant enrichment of the UPR-high program in male donors, consistent with the higher epidemiological prevalence of PD in men, the biological mechanism underlying this sex-specific glial response remains unexplored in this study. Whether it reflects hormonal influences, sex-chromosome-linked gene regulation, or epigenetic differences in glial cells is an open question that future work should address. Together, these constraints underscore the need to investigate how brain-wide glial synchrony is organized spatially and whether regional microenvironments dictate how these programs are expressed.

In summary, our results offer a detailed atlas of glial cell types in the dorsal striatum and establish a model of PD characterized by distinct, non-overlapping multicellular programs. These differences are likely to contribute to variability in clinical presentation and disease progression. Critically, the brain-wide intra-donor consistency of these signatures opens a concrete avenue toward biomarker development that may be accessible through CSF or peripheral immune profiling. Transcription factors like *HSF1* to detect the UPR response and *STAT1/2* for the reactive signature could offer a route to patient stratification without requiring post-mortem tissue. Furthermore, the independence of the UPR-high program from local Lewy body burden observed even in regions devoid of pathology at intermediate Braak stages suggests that this glial state precedes or operates in parallel to canonical neuropathological progression. Integrating these molecular signatures with clinical and genetic data will be key to defining a biologically grounded classification of PD, guiding more precise diagnostic and therapeutic strategies tailored to the underlying glial state of each patient.

## Methods

### Human Tissue

This study is anchored in the analysis of high-quality postmortem human brain tissue. We selected donors based on a strict alignment of clinical history and postmortem neuropathological confirmation. The study consists of autopsy-confirmed Parkinson’s disease cases and control donors free from clinical or pathological signs of neurodegeneration. To build this cohort, we coordinated with three major biobanks: the Human Brain and Spinal Fluid Resource Center (Los Angeles, CA, USA), the Parkinson’s UK Brain Bank (London, UK), and the Massachusetts Alzheimer’s Disease Research Center (Charlestown, MA, USA). For anatomical consistency, we performed targeted dissections of the Caudate nucleus and Putamen, specifically harvesting tissue from coronal slabs aligned at the level of the nucleus accumbens. The control group includes 20 individuals (Pu: n=20; CN: n = 18) aged 25 to over 90 years. The PD group is larger, comprising 36 donors (Pu: n = 35; CN: n = 34), all aged between 60 and 90+ years. All tissue was procured under strict ethical guidelines; written informed consent was obtained from donors or their next of kin, and the study protocols were approved by the respective Institutional Review Boards. (For clinical details, see Extended Data Table 1). We must address a specific demographic constraint. Parkinson’s disease is sexually dimorphic, exhibiting a higher prevalence in men. Consequently, we were unable to construct a perfectly sex-matched cohort. Our final study population includes 16 females and 40 males (Control: 6 females/14 males; PD: 10 females/26 males). We acknowledge that his imbalance limits our statistical power to rigorously evaluate the effect of sex as a biological variable, preventing a deep analysis of sex-specific gene expression patterns.

### Tissue Dissociation

We isolated nuclei from fresh-frozen human brain tissue by adapting the Allen Institute for Brain Science protocol (https://www.protocols.io/view/isolation-of-nuclei-from-adult-human-brain-tissue-eq2lyd1nqlx9/v2) to fit our specific sample needs. To protect the RNA and prevent degradation, we performed every step at 4°C. The first step was thawing 100–150mg of tissue on ice. We then moved the tissue into 2 mL of ice-cold, nuclease-free homogenization buffer. This buffer was composed of: 10 mM Tris (pH 8), 250 mM sucrose, 25 mM KCl, 5 mM MgCl_2_, 0.1 mM DTT, Protease inhibitor cocktail (1×, 50× stock in 100% ethanol, G6521, Promega), 0.2 U/μL RNasin Plus (N2615, Promega), and 0.1% Triton X-100. To homogenize the tissue without damaging the nuclei, we used a glass dounce homogenizer. We applied 20 strokes with a loose pestle, followed by 20 strokes with a tight pestle (357538, Wheaton) to ensure the dissociation was through but controlled. The resulting homogenate was passed through a 70 μm strainer and then a 30 μm strainer to clear out debris. We rinsed the strainers with extra buffer to bring the total volume to 6 mL. After centrifuging the liquid at 900 rcf for 10 minutes, we aspirated the supernatant, leaving roughly 50 μL of buffer over the pellet. We then gently resuspended the nuclei in 200 μL of buffer. To purify the sample, we mixed the suspension 1:1 with 50% iodixanol (OptiPrep Density Gradient Medium, D1556, Sigma) prepared in 60 mM Tris (pH 8), 250 mM sucrose, 150 mM KCl, and 30 mM MgCl_2_. We layered this mixture over 500 μL of 29% iodixanol in a 1.5 mL tube and centrifuged it at 13,500 rcf for 20 minutes. We carefully removed the supernatant and resuspended the pellet in 50 μL of cold blocking buffer (1× PBS, 1% BSA, and 0.2 U/μL RNasin Plus). The final volume was adjusted to 500 μL to prepare for sorting. To target neurons specifically, we incubated the samples with 1 μL of PE-conjugated anti-NeuN antibody (1:500, Millimark mouse anti-NeuN PE conjugated, FCMAB317PE, Merck) for 30 minutes on ice, kept in the dark. We then pelleted the nuclei at 400 rcf for 5 minutes, removed the supernatant, and resuspended them in 500 μL of blocking buffer. Right before fluorescence-activated nuclei sorting (FANS), we filtered the suspension through a 20 μm mesh and added 1 μL of DAPI (0.1 mg/mL, D3571, Invitrogen) for staining.

### Fluorescent-activated nuclei sorting (FANS)

We conducted the FANS at 4°C, using either a BD FACSAria Fusion or a BD FACSAria III flow cytometer. Throughout the sorting process, the nuclei suspension was shielded from light to prevent any loss of fluorescence. By gating the nuclei based on their DAPI and phycoerythrin signals, we were able to distinguish and separate two specific groups:

- NeuN-positive (NeuN^+^): Neuronal nuclei.
- NeuN-negative (NeuN^−^): Non-neuronal nuclei.

We collected each fraction into tubes already prepared with 50 μL of blocking buffer. The sorting continued until we reached a target of approximately 200,000 nuclei for each population. Once collection was finished, we concentrated the nuclei by centrifuging them at 400 rcf for 4 minutes. We then aspirated the supernatant, being careful to leave about 30 μL of buffer behind so we could safely resuspend the pellet. Throughout the remainder of the process, we kept the sorted nuclei on ice to maintain their stability for subsequent analysis.

### Library Preparation

We used the Chromium Next GEM Single Cell 3′ Reagent Kit v3.1 from 10X Genomics (PN-1000268) to build our cDNA libraries from the sorted nuclei. Before starting the main protocol, we manually counted the nuclei and adjusted their concentration to stay within a range of 200 to 1700 nuclei/μL. This step was vital for maximizing capture efficiency. We then introduced the reverse transcription reagents, following the specific 10X Genomics guidelines (CG000204 Rev D, 10X Genomics). We approached the partitioning in two ways using the Chromium Next GEM Chip G (PN-1000120, 10X Genomics). In some instances, we loaded the nuclei fractions into separate lanes, aiming to recover about 5,000 nuclei for each. In other cases, we combined the populations before loading. For these mixed samples, we used a ratio of 70:30 (NeuN^+^:NeuN^−^) and targeted a recovery of 5,000 to 7,000 nuclei. We completed the remaining steps (cDNA amplification and final library construction) using the Single Index Kit T Set A (PN-1000213, 10x Genomics) as instructed by the manufacturer. To make sure the libraries would perform well during sequencing, we conducted several quality control checks:

- Size Distribution: We used the Agilent High Sensitivity DNA Kit (5067–4626, Agilent Technologies) to check the fragment sizes.
- Quantification: We determined the final concentration of the libraries using the KAPA Library Quantification Kit (2700098952, Roche).

These steps allowed us to confirm that the libraries were properly prepared and accurately measured before we proceeded to the sequencing stage.

### Illumina Sequencing

Indexed libraries were multiplexed prior to sequencing, with pools comprising up to 19 samples when targeting a recovery of 5,000 nuclei per sample, or up to 16 samples when aiming for 7,000 nuclei. The pooled libraries were sequenced on an Illumina NovaSeq 6000 system using an S4–200 (v1.5) flow cell across eight lanes. A 28-8-0-91 base pair read configuration was applied, consistent with the requirements of the 10X Genomics 3′ gene expression workflow. Sequencing services were provided by the National Genomics Infrastructure (NGI) in Stockholm, Sweden.

### snRNA-seq Data Analysis

#### Pre-Processing

Raw sequencing output from the single-nucleus RNA-seq experiments was converted into gene-by-cell count matrices using the Cell Ranger pipeline (v3.0.0, 10x Genomics). Reads were mapped to the human reference genome hg38 (GRCh38.p5; NCBI accession GCA_000001405.20). Given the nuclear origin of the RNA, the alignment strategy accounted for both exonic and intronic reads, thereby capturing the full spectrum of nuclear transcripts and improving quantification accuracy.

#### Quality-Control

The first step in our sequencing data was to identify potential doublets using Scrublet^55^. To ensure our results were consistent and reproducible, we ran the tool independently on each sample for 100 iterations, using a fixed random seed and automated thresholds. We then removed any nuclei that were flagged as doublets in more than 10% of those runs. Next, we set specific quality filters based on the distribution of unique molecular identifiers (UMIs) and gene counts. We excluded nuclei that fell below 500 UMIs or 1,200 genes, as these often represent poor-quality samples. On the high end, we removed those with more than 250,000 UMIs or 15,000 genes to avoid suspected multiplets. We also discarded any nuclei where mitochondrial transcripts made up more than 10% of the total content.

To further refine the data, we used a second-degree polynomial regression to model the relationship between log-transformed UMI counts and gene counts. If a nucleus deviated from this predicted curve by more than 2,000 counts, we classified it as an outlier and removed it. To catch more subtle doublets (specifically those involving different cell types), we checked the expression of marker genes for major brain classes, including neurons, astrocytes, and microglia.

- We calculated a cell-type score for every nucleus based on the average expression of these markers.
- Because these scores showed a bimodal distribution, we modeled them using Gaussian mixture distributions.
- We set a strict threshold at the mean of the lower component plus four standard deviations.

Any nucleus that scored above this threshold for more than one cell class was considered a doublet and was excluded. Finally, we wanted to ensure our data reflected the intended brain region. We looked for regional marker genes identified in the Allen Brain Atlas, such as *NEUROD2* and *SLC17A7*. We removed nuclei expressing markers associated with neighboring areas like the claustrum or amygdala to prevent contamination.

#### Glia and non-neuronal detection

Given that the primary focus of this work is on glial and other non-neuronal cell populations, our first analytical step was to clearly distinguish these cells from neuronal subtypes within the dataset. After normalization, we selected 2,500 highly variable genes (HVGs), which were used for downstream dimensionality reduction and clustering analyses.

Principal component analysis (PCA) was performed, and the top 15 principal components were selected to capture the most informative sources of transcriptional variation. To account for potential batch effects arising from differences in sample processing, we applied Harmony for data integration and correction. Using the corrected embeddings, we constructed a neighborhood graph in Scanpy^56^ with 15 nearest neighbors and generated an initial UMAP projection to visualize the global organization of cells in low-dimensional space.

Cell clustering was performed using the Louvain algorithm, enabling the identification of transcriptionally distinct populations. Clusters were subsequently annotated based on the expression of well-established marker genes: OPCs (*PDGFRA*, *VCAN*), Oligodendrocytes (*MBP*, *PLP1*), Astrocytes (*GFAP,ALDH1L1,S100B*), Ependymal Cells (*FOXJ1*, *CCDC153*), Microglia (*CSF1R, CX3CR1, P2RY12*), Perivascular Macrophages (*CD163, MRC1*), Vascular Cells (*CLDN5,PECAM1*), Fibroblasts (*ABCA9,BICC1,COL12A1*), Lymphocytes (*PTPRC,CD3D,CD3G,CD3E*), Medium Spiny Neurons (*NGEF,PDE1B*), and Interneurons (*LHX6*). Following this classification, we subsetted the dataset to retain only glial and non-neuronal populations. This curated dataset formed the basis for all subsequent analyses.

#### Subclustering of glial and non-neuronal populations

We used a consistent analytical approach to subcluster all glial and non-neuronal lineages, ensuring that our results were comparable across different populations. Once we had our major cell classes (including astrocytes, microglia, vascular cells and lymphocytes), we focused on the 2,000 top variable genes (HVGs) in each group. We then ran PCA and kept the first 15 principal components, as these provided the most relevant information regarding the transcriptional differences within each lineage.

To account for technical differences between sequencing batches, we used the Harmony algorithm on each cell population. Using the corrected data, we built neighborhood graphs and identified clusters with the Louvain algorithm. We started with a base resolution of 0.2 but adjusted this for each lineage to capture the most meaningful biological details. In cases where a single cluster clearly contained distinct biological groups, we performed additional subclustering to separate them.

To figure out what each subcluster represented, we looked for genes that were expressed differently between subclusters. We used the scanpy.tl.rank_genes_groups function with a Wilcoxon rank-sum test. We specifically looked for candidate markers that met two criteria: a log fold change greater than 0.5, and an adjusted p-value of less than 0.05. We then manually reviewed these top genes to assign a biological identity to each subcluster. The full list of these marker genes and the resulting subpopulation names can be found in supplementary tables.

#### Hierarchical Analysis

To better understand how the different subpopulations relate to one another transcriptomically, we used the scanpy.tl.dendrogram function within the Scanpy framework. We measured the similarity between clusters by calculating Pearson correlation coefficients based on their gene expression profiles. For the hierarchy itself, we chose the complete linkage criterion. This method determines the structure by looking at the maximum distance between elements in different clusters when building higher-order groups.

#### Compositional Analysis

When comparing how subpopulation sizes changed between conditions, we had to address the fact that single-cell data is compositional. Since the cell-type percentages in any given sample must always add up to 100%, looking at raw percentages alone can be misleading results or render false associations. To solve this, we applied a centered log-ratio (CLR) transformation to our subpopulation proportions before running any statistical tests. Instead of looking at raw numbers, this method calculates the abundance of each subpopulation relative to the geometric mean of all populations in that same sample. This step is crucial because it removes the “constant-sum” constraint, making our comparisons statistically sound. Once the data was transformed, we used the Mann-Whitney U test to compare the values between different conditions.

#### Module Analysis

To uncover coordinated gene programs within glial subpopulations, we applied the Hotspot algorithm to the astrocyte and microglial datasets. This approach identifies modules of co-expressed genes by leveraging structured patterns within single-nucleus transcriptomic data, thereby highlighting groups of genes that are likely to participate in shared biological processes.

Analyses were conducted using default parameters, while restricting the input to the 10,000 top HVG in each lineage. This filtering step allowed us to concentrate on the most informative transcriptional features and reduce background noise. Through this strategy, we aimed to delineate gene modules that define functional heterogeneity within astrocytes and microglia and to better understand the molecular programs shaping their behavior in the context of disease.

#### Differential Expression Analysis

To identify transcriptional alterations associated with PD, we performed differential expression analysis comparing PD and Control samples for astrocytes and microglia separately. We considered all cells within each population rather than stratifying by subpopulation at this stage. This approach allowed us to capture global disease-associated transcriptional shifts at the level of the entire cell class.

Differential gene expression was computed using the sc.tl.rank_genes_groups function implemented in Scanpy, applying the Wilcoxon rank-sum test to compare nuclei labeled as “PD” against those labeled as “Control”, and using Benjamini-Hochberg’s correction for multiple hypotheses testing. Genes were classified as upregulated or downregulated in PD based on statistical significance and effect size criteria. To further interpret the biological relevance of these changes, we intersected the lists of differentially expressed genes with previously defined marker genes for astrocyte and microglial subpopulations. This overlap analysis enabled us to assess whether PD-associated transcriptional alterations preferentially corresponded to specific cellular states or subtypes within each lineage. By integrating global differential expression with subpopulation-specific signatures, we aimed to determine whether disease-related changes reflect broad shifts across the entire lineage or are driven by particular reactive or functionally distinct subpopulations.

#### KEGG PD Analysis

The aim of this analysis was to determine if genes linked to PD were more active in specific astrocyte or microglial subpopulations. To keep our findings relevant to the disease itself, we focused this enrichment analysis strictly on nuclei derived from PD samples. This allowed us to map out how disease-related molecular programs are distributed across different glial states within a pathological environment.

We started by gathering a list of PD-related genes from the KEGG database^28^. Using the sc.tl.score_genes function in Scanpy, we calculated a module score for every cell. This score represents the combined expression level of the KEGG PD gene set, resulting in a quantitative look at how strongly each cell is running the PD-associated transcriptional program.

To determine if specific subpopulations were significantly enriched with this PD signature, we compared the scores of cells in one cluster against all other cells using a one-versus-rest approach. We then measured differences using Wilcoxon rank-sum test to evaluate these differences, determined the mean scores for each group and calculated the logfoldchanges, and controlled for errors by adjusting our p-values using the Benjamini-Hochberg’s false discovery rate (FDR) procedure. This process helped us identify which glial populations were most involved in the PD-related gene activity.

#### Transcription Factors Analysis

Gene regulatory network activity was inferred using the SCENIC workflow^57^. Briefly, regulons (transcription factors and their predicted target genes) were identified and regulon activity per cell was quantified using the AUCell algorithm, generating an AUC matrix stored in the AnnData object. Downstream analyses were performed on regulon activity scores rather than gene expression levels.

To investigate regulatory programs associated with biological responses (Control-like, UPR, and Reactive), cells were grouped according to annotated glial subpopulations. For each subpopulation, regulon activity was compared against relevant reference subpopulations using pairwise statistical testing. Regulons significantly upregulated in one subpopulation relative to others were identified and ranked based on effect size statistics.

Two complementary strategies were implemented:

- Response-shared regulons: regulons consistently upregulated across subpopulations belonging to the same response category were identified by intersecting significant regulons across the corresponding subpopulations. This approach aimed to capture regulatory programs commonly associated with a shared biological response, independent of cell-type identity.
- Subpopulation-specific regulons: regulons preferentially enriched in individual subpopulations were identified by comparing each subpopulation against related subpopulations within the same lineage. Regulons uniquely enriched in a given subpopulation were defined as those significantly upregulated in that subpopulation and not enriched in others. This strategy was designed to highlight regulatory programs underlying subtype-specific identity or state transitions.

#### Cell-Cell Interaction Analysis

Cell-cell communication analysis was performed using the LIANA framework^43^ to infer putative ligand-receptor interactions between annotated glial subpopulations. We applied the rank_aggregate method, which integrates multiple ligand-receptor resources into a consensus score to provide robust interaction estimates. LIANA was run on the full single-cell glia and non-neuronal dataset using cell subpopulation annotations, the consensus resource, a minimum expression proportion threshold of 0.3 (expr_prop = 0.3), and a minimum of 100 cells per interacting group (min_cells = 100). Differential expression was assessed using the Wilcoxon rank-sum test.

The resulting interaction table provides ligand-receptor pairs with associated interaction strength (LR score) and specificity rank. For downstream analyses, we specifically focused on bidirectional communication between the AR2 astrocyte subpopulation and the M1 microglial subpopulation. Interactions were therefore restricted to those in which AR2 acted as sender and M1 as receiver, or vice versa, preserving signaling directionality. To obtain robust estimates, interaction scores were aggregated across observations to compute mean LR scores and mean specificity ranks for each ligand-receptor pair. To prioritize high-confidence and biologically meaningful interactions, we applied stringent filtering criteria, retaining only interactions with high mean specificity rank and high mean LR score. These filtered interactions were subsequently used for visualization and interpretation of asymmetric signaling patterns between AR2 and M1 reactive glial states.

#### UPR Signaling Analysis

To characterize the activation status of the Unfolded Protein Response (UPR) across cell subpopulations, we performed a dot plot analysis using the Scanpy library. We examined the expression of specific marker genes representing the three canonical UPR branches: the IRE1 arm (*XBP1,TRAF2,MAPK8,MAPK14*), the ATF6 arm (*ATF6,MBTPS1,MBTPS2*), and the PERK arm (*EIF2AK3,EIF2S1,ATF4*). This analysis was conducted across eleven identified subpopulations: A0, AR1, AR2, MH, MH_HSP, M1, MOL-A, PDAO-1, PDAO-2, OPCs-A, and PDAOPCs. In the visualization, the dot size represents the fraction of cells within each subpopulation expressing the gene of interest, while the color intensity corresponds to the mean expression level within the expressing fraction.

#### Braak Lewy Body Staging Analysis

To assess changes in cellular composition across Braak Lewy Body (LB) stages while accounting for the compositional nature of single-cell data, we applied a centered log-ratio (CLR) transformation at the subject level. For each subject, cells were first grouped by subpopulation and raw counts were converted into a composition vector representing the relative abundance of each subpopulation. A pseudocount of 1 was added to avoid undefined logarithms for zero counts. The CLR transformation was then computed by dividing each subpopulation count by the geometric mean of all subpopulation counts within the same subject and taking the natural logarithm. This approach normalizes each subject’s cellular composition relative to its overall cellular landscape, thereby mitigating biases introduced by differences in total cell numbers and ensuring that subpopulation abundances are interpreted in relation to one another rather than independently.

Braak LB-stage-level summaries were obtained by averaging subject-level CLR values within each stage. To avoid spurious effects driven by single donors, subpopulation-stage combinations represented by fewer than two subjects were excluded from stage-level summaries. For visualization, CLR values were optionally standardized (z-scored) across Braak LB stages for each subpopulation to facilitate comparison of relative enrichment or depletion trends. This compositional framework enables detection of subpopulation-specific increases or decreases across Braak LB stage progression while appropriately accounting for interdependence among cell-type proportions.

#### Correlations between glial subpopulations and Lewy Body quantifications

To examine the relationship between glial subpopulation abundance and α-synuclein aggregations, for this analysis we restricted the data to the subset of donors for whom quantitative immunohistochemical measures of LB density (LBs/Area) were available, as previously reported in Barba-Reyes *et al*^14^. Analyses were performed separately for Pu and CN, and donors contributing fewer than 50 nuclei in a given region were excluded to ensure reliable compositional estimates.

For each remaining donor, subpopulation counts were computed and converted to proportions of the total nuclei recovered from that region. These proportions were subsequently CLR-transformed as described above to account for the compositional structure of the data. Spearman rank correlations were then calculated between the CLR-transformed abundance of each subpopulation and the corresponding regional LB density, independently for Pu and CN. A predefined set of disease-relevant subpopulations was selected for this analysis: A0, AR1, AR2, MH, MH-HSP, M1, DAM, OPCs-A, PDAOPCs, MOL-A, PDAO-1, PDAO-2, and PDAO-3.

#### Donor stratification based on spatial analysis’ priors

To investigate how cellular composition varies across the cohort, we performed patient stratification based on the proportional representation of specific astrocyte subpopulations. The selection of subpopulations used for stratification (AR1 and AR2) was informed by our prior sample representation analysis done using only the grey matter profiled by the Xenium datasets, which identified these clusters as key indicators of pathological diversity. Stratification was performed by calculating the relative abundance of each subpopulation within individual samples. SVZCs were not considered when computing relative abundance as they were found to be specific to the lateral ventricle. Patients were classified into distinct clinical groups using a hierarchical thresholding approach where samples were first evaluated for their AR1 (Unfolded Protein Response-related) astrocyte population. Any sample where AR1 exceeded 10% of the total astrocyte composition was assigned to the UPR group. For the remaining samples, those where the AR2 (Reactive) astrocyte population exceeded 10% were classified as Reactive. Samples that did not meet the minimum 10% threshold for either AR1 or AR2 were categorized as Homeostatic. This stratification allowed us to correlate specific transcriptomic states identified in the single-cell data with broader patient-level pathological profiles.

#### Differential expression analysis and comparison between donor groups and PD

To evaluate to which extent differentially expressed genes in PD were driven by donor groups’ multicellular programs, we performed differential expression analysis between groups for each cell type annotated. Differential expression testing was conducted using scanpy's sc.tl.rank_genes_groups function, employing the Wilcoxon rank-sum test.

Two distinct analytical frameworks were employed. First, state-specific comparison where donors representing UPR and Reactive donor groups were tested against a baseline of homeostatic donors, intentionally excluding cells from the reciprocal PD-associated donor group from the subset to maintain reference purity. In addition, we performed differential expression analysis between cells obtained from donors with PD and healthy controls. Significant differentially expressed genes (DEGs) were defined by an adjusted p-value of less than 0.05 and a minimum expression prevalence of 10% (pts > 0.1). To characterize the magnitude of transcriptional changes, these genes were categorized into four mutually exclusive tiers based on the absolute value of their log2 fold-change (LFC): Tier 1 (LFC ≥ 2.0), Tier 2 (1.0 ≤ LFC < 2.0), Tier 3 (0.5 ≤ LFC < 1.0), and Tier 4 (0.25 ≤ LFC < 0.5). The distribution and directionality of these genes were visualized using stacked bar charts, with up-regulated and down-regulated genes plotted on opposing y-axes and color-coded by tier.

To assess transcriptional overlap between cellular states and disease conditions, three-way Venn diagrams were generated for each cell type using the matplotlib-venn library. Potential pseudogenes and non-coding transcripts, identified by prefixes such as AC, AL, LINC, AP, or AF, were filtered out prior to analysis. A dual-threshold strategy was employed to identify shared signatures: a candidate universe of genes was first established using a strict threshold (e.g., |log_2_fold-change| ≥ 1.0 and *P*_*adj*_ < 0.05). in at least one condition, and their presence in the remaining conditions was evaluated using a relaxed threshold (e.g.,|log_2_fold-change| ≥ 0.5 and *P*_*adj*_ < 0.05). This methodology allowed for the identification of genes with consistent directionality across groups even when the magnitude of change varied.

To evaluate gene sharing across different cell types for each condition, UpSet plots were generated using the upsetplot library. This analysis utilized a the same logic used for identified shared DEG between multicellular programs and PD, where a candidate universe of genes was first established for each comparison (UPR, Reactive, or PD) using a strict threshold (|log_2_fold-change| ≥ 1.0 and *P*_*adj*_ < 0.05). The presence of these candidate genes was then cross-referenced across all other cell types using a relaxed threshold (|log_2_fold-change| ≥ 0.25 and *P*_*adj*_ < 0.05). In plots, only intersections containing more than 10 genes were visualized.

#### Functional Enrichment Analysis

To characterize the biological functions associated with unique and shared gene sets across donor groups, we performed gene set enrichment analysis (GSEA) using the GSEApy (v1.1+) library. For each cell type, we previously partitioned differentially expressed genes into seven distinct overlap categories: genes unique to PD, Reactive, or UPR states, and genes shared between specific combinations (e.g., PD + Reactive, PD + UPR, and those common to all three). This partitioning was achieved by intersecting gene sets defined by our previously established strict and relaxed thresholds.

Functional enrichment was conducted using the GO Biological Process 2023 database. For each category containing at least five genes, the top three most significant terms (based on adjusted p-values) were selected to define a master pathway list for each cell type and visualized using comparative dotplots, where the dot size represents the gene ratio (proportion of the gene set overlapping with the GO term) and the color intensity reflects the significance level expressed as −log10(Adjusted p-value).

### Spatial transcriptomics (Xenium) data generation

#### Spatial transcriptomics platform

The Xenium datasets analyzed in this study included both previously published datasets^14,45^ and newly generated datasets. For the newly generated datasets, spatial transcriptomic data were obtained using the Xenium in situ platform (10X Genomics), enabling subcellular-resolution characterization of RNA within tissue sections, consistent with the approach described in Garma *et al*.^45^ and Barba-Reyes *et al*^14^

#### Gene panel design

The Xenium technology uses oligonucleotide probes to measure gene expression from a predefined panel. In this study, we employed a gene panel consisting of 266 genes from the Xenium Human Brain Gene Expression Panel. An additional 100 genes were selected based on our snRNA-seq dataset, resulting in a custom panel (Xenium Custom Gene Expression Panel 51–100, Z3DREH, PN-1000561, 10X Genomics).

#### Experimental workflow

Tissue blocks from human subjects (N = 4; including putamen and caudate nucleus) were retrieved from −80 °C storage and transported on dry ice to a cryostat (CryoStar NX70, Thermo Scientific). Samples were mounted on the specimen holder using Tissue Tek O.C.T. Compound (4583, Sakura) and equilibrated to −20 °C within the cryostat chamber for 5 minutes. Tissue sections of 10 μm thickness were collected and placed directly into the imaging region of precooled Xenium slides (12×24 mm, PN-3000941, 10X Genomics). Section adherence was facilitated by briefly warming the reverse side of the slides with gentle pressure, followed by immediate refreezing on the cryobar. Slides with mounted tissue were kept in the cryostat during sectioning and subsequently stored at −80 °C.

Downstream processing (including probe hybridization, ligation, and rolling circle amplification) was performed at the in situ Sequencing Infrastructure Unit (Science for Life Laboratory, Stockholm) according to the manufacturer’s protocol (CG000582 Rev E, 10X Genomics), with chemical quenching applied to minimize background fluorescence. Tissue sections were imaged using the Xenium Analyser (10X Genomics), which also performed signal decoding and data acquisition. This same protocol was used to generate previously published datasets included in^14,45^.

### Spatial transcriptomics (Xenium) data analysis

#### Segmentation, data preprocessing and cell type identification

Xenium experiments provide spatial positions for all decoded reads. Therefore, the first step in processing these datasets was to segment individual cells and determine their transcriptomic composition. In this study, cells were defined using the default nuclear Xenium segmentation, a conservative approach that minimizes the risk of misse^23^ention21. The segmented cells were preprocessed using Scanpy^56^. Preprocessing involved filtering out low-quality cells, removing those with fewer than 50 total counts or fewer than 5 detected genes. The data were subsequently normalized to total counts, log-transformed, and scaled. We performed PCA and constructed a neighborhood graph (n_neighbors=10, n_pcs=30) to generate a UMAP embedding for visualization. Leiden clustering at a resolution of 2.0 was applied to identify distinct cell populations, which were then manually annotated using an external reference based on marker gene expression and spatial position. For subpopulation identification, each population was subclustered and manually annotated based (Code availability).

#### Gene module analysis using Hotspot

To identify spatially or transcriptionally coherent gene modules in the Xenium datasets, as we did in snRNA-seq, we employed the Hotspot algorithm. Analysis was initiated using a Depth-Adjusted Negative Binomial (DANB) model to account for technical variation and library size differences, using the total counts per cells for UMI normalization. A K-Nearest Neighbors (KNN) graph was constructed using the top 20 neighbors in the PCA latent space to define the local cellular environment.

We evaluated the significance of gene expression patterns by calculating local autocorrelations (Geary’s C) across the KNN graph. Genes showing non-random spatial or local distribution were filtered using a False Discovery Rate (FDR) threshold of 0.05. To group these informative genes, a local correlation matrix was computed, capturing pair-wise gene relationships within local neighborhoods.

Modules were subsequently defined using a minimum threshold of 5 genes per module and a clustering FDR of 0.01. For each identified module, we calculated per-cell scores to represent the aggregate local expression of the module's gene members. These scores were integrated into the dataset to facilitate downstream analysis of biological programs across the identified sample regions. To facilitate their interpretation, manual annotation of the gene modules was done based on the exploration of the module’s composition and scores across cells

#### Niche identification

NicheCompass^25^ was applied to delineate cellular domains by integrating spatial transcriptomics data across all samples. A latent graph embedding was generated using a graph convolutional network (GCN) model, incorporating categorical covariates such as sample replicates. The model was trained for 400 epochs with regularization to enhance ligand–receptor–based niche identification. Clustering of the latent embeddings was then performed using the Leiden algorithm (with a resolution of 0.2), and the resulting niches were annotated according to their spatial distribution and cellular composition.

#### Identification of spatially-variable genes

To identify spatially variable genes (SVGs), we performed a sample-wise spatial autocorrelation analysis using the Squidpy framework^58^. Since we focused on the grey matter regions, for each sample, we kept only cells identified as part of the grey matter during domain analysis. Next, spatial connectivity was modeled via Delaunay triangulation to define cell neighborhoods. Within each sample, we calculated Moran’s I statistics to quantify global spatial patterns, assessing significance through 100 permutations per gene. To ensure robust estimation, analysis was restricted to samples with at least 50 observations. The resulting Moran’s I scores and p-values were aggregated across all replicates, enabling a comparative assessment of spatial transcriptomic signatures across experimental conditions.

#### Sample representation of spatial datasets

To analyze high-level variation across donors and identify distinct disease endotypes in the Xenium samples, we performed sample-level representation learning using the patpy^38^ implementation of the GloScope^37^ method. For this, we selected only cells identified within the striatal gray matter domain and we excluded samples which were found a posterior to present a hybrid molecular profile, being classified as neurological controls, but presenting a Braak stage > 0. Before representation, we consider sample filtering for those samples containing less than 500 cells, with no samples excluded. Sample representations were generated using the GloScope algorithm, which models each donor as a distribution of cells within the X_{pca} latent space. We calculated a sample-to-sample distance matrix by estimating the divergence between these cell-state distributions using a k-nearest neighbor (k=6) approach, resulting in an embedding where samples with similar cellular compositions cluster together.

The resulting distance matrix was used to construct a sample-level neighbor graph, which was visualized via UMAP with a minimum distance of 0.02 to preserve local structure. Donor subgroups, or endotypes, were identified using Leiden clustering at a resolution of 0.9. After group annotation based on molecular and cell type composition, we integrated our analysis with complementary comprehensive clinical and technical metadata, including Braak and Thal stages, Post-Mortem Interval (PMI), Age, and Sex, while technical variables such as median nucleus area and total counts per sample were monitored for potential batch effects.

## Supplementary Files

This is a list of supplementary files associated with this preprint. Click to download.

- SupplementaryTablesLegend.pdf
- SupplementaryFigures.pdf
- UnifiedExtendedDataTables.xlsx

## Data Availability

All code corresponding to the analysis of single-nuclei RNAseq dataset is available at figshare and the code corresponding to spatial transcriptomics (Xenium) datasets is available at https://github.com/Moldia/PD_striatal_xenium.

## Data availability

Raw and normalized counts from single-nuclei RNA-seq data are available at figshare. Processed and annotated cells from Xenium experiments, in the form of AnnData, as well as a KaroSpace viewer can be downloaded from the following Zenodo repository. These data will be publicly available as of the date of publication.
